# Emergence of methicillin-resistant *Staphylococcus aureus* from clonal complex 398 with no livestock association in Brazil

**DOI:** 10.1590/0074-02760170040

**Published:** 2017-09

**Authors:** Egidio Domingos André, Renata Freire Alves Pereira, Robert Eugene Snyder, Thamiris Santana Machado, Lialyz Soares Pereira André, Claudete Aparecida Araújo Cardoso, Fábio Aguiar-Alves

**Affiliations:** 1 Universidade Federal Fluminense, Laboratóro Universitário Rodolfo Albino, Laboratório de Epidemiologia e Biotecnologia Molecular, Niterói, RJ, Brasil; 2 Universidade Federal Fluminense, Faculdade de Medicina, Departmento Materno-Infantil, Niterói, RJ, Brasil; 3 Universidade Federal Fluminense, Instituto de Biologia, Programa de Pós-Graduação em Ciência e Biotecnologia, Niterói, RJ, Brasil; 4 University of California, School of Public Health, Division of Epidemiology, Berkeley, California, USA; 5 Universidade Federal Fluminense, Escola de Medicina, Programa de Pós-Graduação em Patologia, Niterói, RJ, Brasil

**Keywords:** MRSA, CC398, genotyping

## Abstract

CC398 is a livestock-associated *Staphylococcus aureus*. However, it has also been isolated from humans with no previous contact with livestock. A surveillance of methicillin-resistant *S. aureus* colonisation among children attending public day care centres and hospitals in Niterói and Rio de Janeiro, Brazil, between 2011 and 2013, resulted in the isolation of six cases of CC398 from individuals with no previous exposure to livestock. These isolates showed a high frequency of the *erm*(C) gene (4/6, 66.7%) with induced resistance to clindamycin, and a relatively high frequency of *SE*s and *lukS/lukF* genes. These results suggest the emergence of a non-LA-CC398 in Brazil.

*Staphylococcus aureus* colonisation is the main risk factor for subsequent infection, and this risk is higher if the staphylococci are methicillin-resistant (MRSA) (Safdar & Bradley 2008). Children experience particularly high rates of morbidity and mortality from staphylococcal infections (Iwamoto et al. 2013).

Clonal Complex 398 (CC398) is among the most important livestock-associated (LA) *S. aureus* genotypes, and contact with animals is a well-described risk factor for its colonisation or infection in humans (Smith & Wardyn 2015). However, recent studies have described a non-LA-CC398 lineage in humans with no previous contact with livestock (Uhlemann et al. 2012, Larsen et al. 2015, Smith & Wardyn 2015). This lineage has rapidly spread in humans over a wide-ranging geographical area, in particular in Europe and North America, and has been linked to invasive infection where it has been reported. These *S. aureus* isolates usually carry *spa* type t571 or t1451 (Valentin-Domelier et al. 2011, Uhlemann et al. 2012, Larsen et al. 2015, Smith & Wardyn 2015).

In Brazil, as in all of Latin America, formal, integrated, or systematic *S. aureus* epidemiological surveillance systems are almost universally absent. To date, only three cases of CC398 have been reported in Brazil. The first was an MRSA isolate from a healthy dairy cow (staphylococcal protein A type t011) from a farm in the state of São Paulo (Silva et al. 2014), the second was an MSSA isolate that led to fatal pneumonia in a cancer patient in São Paulo (t034) (Gales et al. 2015) and the most recent was a case of an MRSA that was isolated from a patient with cystic fibrosis who had previous contact with farm animals (Lima et al. 2017). In South America, outside of Brazil, CC398 has also been reported in Colombia, also resulting in the death of the patient (Jiménez et al. 2011), and Peru, where it was isolated from several pigs at a commercial farm (Arriola et al. 2011).

Here, we report six cases of nasal colonisation by MRSA CC398 among children without any previous exposure to traditional CC398 risk factors (living in or having recently travelled to rural areas, or having recent contact with farm animals or livestock) between August 2011 and March 2016 in the cities of Niterói and Rio de Janeiro, Brazil.

The six CC398 bacterial isolates were identified from nasal swabs of 1852 children sampled in four different settings, including both outpatient and inpatient clinics, and 23 daycare centres. Four CC398 isolates were identified from a sampling of 500 children at two public inpatient clinics, while two were identified among 852 children reporting to paediatric outpatient clinics at two public hospitals. There were no children colonised with CC398 among the 500 sampled at 23 different daycare centres. All children attending the study sites during the period, whose parents agreed to the terms, were included in the study.

A total of 1852 nasal swab samples were collected in the cities of Niterói and Rio de Janeiro between 2011 and 2016 from children in five independent MRSA surveillance studies, in day care centres and public hospitals. The samples were submitted for laboratory testing of catalase and coagulase, plating in mannitol, antibiogram generation and for polymerase chain reaction (PCR) of the *mec*A gene, to identify MRSA samples. All 179 identified MRSAs were genotyped by PCR, multiplex-PCR (Oliveira & de Lencastre 2002), *spa*-Typing (staphylococcal protein A typing) (Shopsin et al. 1999) and Multi-locus sequence type (MLST) methods (Enright et al. 2000). Six were identified as ST398 by MLST. Five had the t1451 *spa* type and one was t1985. Five carried the SCC*mec* IV and one the SCC*mec* V. All six CC398 strains were non-typeable by PFGE with *Sma*I, corroborating previous reports (Larsen et al. 2015, Smith & Wardyn 2015). The species specificity of all isolates was confirmed by a positive PCR reaction for both the *SA442* and *fem*A genes.

Antimicrobial susceptibility was performed with 14 antimicrobial disks (oxacillin, cefoxitin, penicillin, ciprofloxacin, chloramphenicol, clindamycin, erythromycin, nitrofurantoin, gentamicin, rifanpicin, trimethoprim, sulfamethoxazole, tetracycline and vancomycin) according to The Clinical and Laboratory Standards Institute guidelines (CLSI 2015), and resistant isolates were subjected to PCR for the identification of resistance genes. Four isolates were resistant to erythromycin (4/6, 66.7%), with all six (100%) demonstrating an induced resistance to clindamycin (D-test positivity) that was encoded by the *erm*(C) gene. One isolate was resistant to trimethoprim/sulfamethoxazole (1/6, 16.7%). This was confirmed by PCR of the *dfr*G gene.

PCR was used to confirm the presence of genes encoding staphylococcal enterotoxins (SEs) (*sea*, *seb*, *sec*, *sed*, *see*, *seg*, *seh*, *sei*, *selj*, *selk*, *sell*, *selm*, *seln*, *selo*, *selp*, *selq*, *selr* and *tst1*) and *lukS/lukF*, encoding Panton-Valentine leukocidin (PVL). All isolates carried multiple SE genes (Table), and three (50%) were PVL-positive (Lina et al. 1999, Omoe et al. 2005).

**TABLE:** Genotype profiles of CC398 methicillin-resistant *Staphylococcus aureus* (MRSA) strains colonising the nares of six children in Rio de Janeiro, Brazil, 2011-2016

Isolate	Location	Date of Collection	Ridom *spa* type	SCC*mec* type	Antimicrobial resistance	Resistance genes	Other genes
AMBU125	Outpatient	7/3/2012	t1451	IV	PEN, OXA, CFO, ERY^D^	*bla*Z, *mec*A, *emr*(C)	PVL, *fem*A, *seg*, *sei*, *selk*, *selm*, *seln*, *selo*, *selq, selr*
HGVF080	Inpatient	9/15/2011	t1451	IV	PEN, CFO, ERY^D^	*blaZ*, *mec*A, *emr*(C)	*fem*A, *seg*, *selk*, *selm*, *seln*, *selo*, *selp, tst1*
HGVF396	Inpatient	7/17/2012	t1451	IV	PEN, CFO	*blaZ*, *mec*A	PVL, *fem*A, *sea*, *seg*, *seh*, *sei*, *selk*, *sell*, *selm*, *seln*, *selo*, *selp*, *selq, selr*
HGVF398	Inpatient	7/19/2012	t1451	IV	PEN, CFO, OXA	*blaZ*, *mec*A	PVL, *fem*A, *seg*, *seh*, *sei*, *selk*, *sell*, *selm*, *seln*, *selo*, *selp*, *selr, tst1*
P1036C	Outpatient	4/22013	t1985	IV	PEN, CFO, ERY^D^, TMP, SMX	*blaZ*, *mec*A *emr*(C), *dfr*G	*fem*A, *seg*, *sei*, *selk*, *selm*, *seln*, *selo*, *selp, selr*
DMC49	Outpatient	19/3/2016	t1451	V	PEN, CFO, OXA, ERY^D^	*blaZ*, *mec*A, *emr*(C)	*fem*A, *seg, selj, selp*

CFO: cefoxitin; ERY^D^: erythromycin with induced clindamycin resistance; OXA: oxacillin; PEN: penicillin; PVL: Panton-Valentine leucocidin; SMX: sulfamethoxazole; TMP: trimethoprim.

All six CC398 carriers resided in the urban centre or suburban periphery of Rio de Janeiro, and none reported any contact with livestock or farm animals.

The isolation of CC398 from individuals without any of the lineage’s traditional risk factors suggests the routine transmission of this lineage in several proximate Brazilian urban areas. The isolates exhibited phenotypic and genotypic characteristics consistent with an emergent non-LA-CC398 MSSA strain that is closely related to invasive human infection (Valentin-Domelier et al. 2011, Larsen et al. 2015), suggesting that CC398 may have arrived as MSSA and acquired SCC*mec* in a healthcare setting. Importantly, there were no CC398 isolates in a (2006) surveillance study of cattle conducted by our group in the same geographic area (Unpublished data). Our study describes molecular virulence and resistance characteristics of several non-LA-CC398 MRSA. The intrinsic virulence features of CC398 MSSA described elsewhere and the presence of the same virulence features in the MRSA isolates described here (which are also antibiotic resistant), highlight the risk of invasive infection by these emergent strains (Valentin-Domelier et al. 2011, Larsen et al. 2015).

Given the history of the rapid spread of non-LA-CC398 in humans (Larsen et al. 2015), the affinity that these isolates have for the acquisition of the SCC*mec* cassette (Smith & Wardyn 2015), and the lack of *S. aureus* epidemiological surveillance in Brazil, these findings suggest that the dissemination of CC398 in the country has been widely underreported. Underscoring this, the isolate was identified among children seeking healthcare for reasons other than the traditional risk factors for colonisation or infection (Safdar & Bradley 2008). We must urgently increase the quality of *S. aureus* epidemiological surveillance in Brazil, and elsewhere in Latin America, to avoid infections with other poorly molecularly and epidemiologically described isolates.
